# Comparative Study of Immunomodulatory Agents to Induce Human T Regulatory (Treg) Cells: Preferential Treg-Stimulatory Effect of Prednisolone and Rapamycin

**DOI:** 10.1007/s00005-020-00582-6

**Published:** 2020-06-12

**Authors:** Michał Janyst, Beata Kaleta, Karolina Janyst, Radosław Zagożdżon, Ewa Kozlowska, Witold Lasek

**Affiliations:** 1 Department of Immunology, Centre of Biostructure Research, Medical University of Warsaw, Warsaw, Poland; 2 Department of Clinical Immunology, Medical University of Warsaw, Warsaw, Poland; 3 Postgraduate School of Molecular Medicine, Medical University of Warsaw, Warsaw, Poland; 4 Department of Immunology, Faculty of Biology, University of Warsaw, Warsaw, Poland

**Keywords:** Treg cells, Prednisolone, Rapamycin, Glatiramer acetate, Inosine pranobex, Atorvastatin

## Abstract

T regulatory (Treg) cells play a critical role in the maintenance of self-tolerance, as well as in inhibition of inflammation and exaggerated immune response against exogenous antigens. They develop in the thymus (tTreg cells) but also may be generated at the peripheral tissues, including tumor microenvironment (pTreg cells), or induced in vitro in the presence of transforming growth factor (TGF)-β (iTreg cells). Since tTreg cells constitute a minor fraction of peripheral blood lymphocytes in physiological conditions, an alternative way to obtain high number of functional Treg cells for therapeutic purposes is their generation in vitro from conventional T cells. In our studies, we compared effectiveness of several pharmacological agents with suggested immunomodulatory effects on Treg development (rapamycin, prednisolone, inosine pranobex, glatiramer acetate, sodium butyrate, and atorvastatin) to optimize Treg-inducing protocols. All but one (atorvastatin) immunomodulators augmented induction of polyclonal Treg cells in cultures. They were effective both in increasing the number of CD4^+^CD25^high^Foxp3^high^ cells and Foxp3 expression. Rapamycin and prednisolone were found the most effective. Both drugs prolonged also phenotypic stability of Treg cells and induced fully active Treg cells in a functional assay. In the assay, prednisolone appeared superior versus rapamycin. The results, on the one hand, may be helpful in planning optimal protocols for generation of Treg cells for clinical application and, on the other hand, shed some light on mechanisms of the immunomodulatory activity of some tested agents observed in vivo.

## Introduction

T regulatory (Treg) cells are a fraction of CD4^+^ T lymphocytes responsible for suppression of immune response (Sakaguchi et al. 2008). Their number is low in neonates but in adults, probably due to immunomodulatory effects of environmental factors, percentage of Tregs is increased (Zahran et al. 2019). Although Treg cells constitute minority of CD4^+^ T lymphocytes in blood, these cells play a crucial role in maintaining homeostasis of the immune system (Whibley et al. 2019). Their functions include: prevention of autoimmune diseases by maintaining self-tolerance, suppression of hypersensitivity reactions, induction of tolerance against dietary antigens, induction of maternal tolerance to the fetus, and induction of tolerance to the allograft (Bocian et al. 2017). Malfunction of Treg cells leads to development of many diseases such as allergies, autoimmune and inflammatory diseases, and many other (Lan et al. 2012; Lee et al. 2017; Martín-Orozco et al. 2017).

According to recent recommendations, Treg cells—in respect of their origin and development—can be divided into three different subpopulations (Abbas et al. 2013). Thymically derived T regulatory cells (tTregs, formerly natural Tregs—nTregs) constitute a fraction of Treg cells developed in the thymus and are engaged in recognition of autoantigens and TCR/MHC interactions of high affinity. Peripherally derived T regulatory cells (pTregs, formerly induced Tregs—iTregs) develop in vivo during suboptimal stimulation of T cell receptor (TCR), but require also additional factors, such as transforming growth factot (TGF)-β (Horwitz et al. 2008; Kretschmer et al. 2005). Expansion of pTregs is related to many physiological or pathological processes. They can arise, for example, following presentation of alloantigens by dendritic cells, after stimulation by metabolites produced by commensal bacteria—to prevent exaggerated immune response against microbiota, and as a result of chronic inflammatory pathologies such as inflammatory bowel diseases (Arpaia et al. 2013). The third fraction of T regulatory cells includes iTregs—in vitro-induced T regulatory cells. They are generated from conventional T cells in vitro in cultures supplemented with TGF-β (Schmitt and Williams 2013; Zheng et al. 2002). All the Treg cells have characteristic phenotype: CD4^+^, CD25^high^, Foxp3^+^ and low expression of CD127 (Liu et al. 2006). They also express CTLA-4, GITR, CCR4 and CD62L markers (Horwitz et al. 2008). There is a general view that tTreg cells are preferentially engaged in blocking autoimmune reactions, while pTreg cells are necessary mainly for prevention of excessive response against exogenous antigens.

Due to a potent suppressive potential, Treg cells can be used in the treatment of diseases in which immune reactions are excessive. Effectiveness of the treatment was documented for the first time in patients with ongoing graft-versus-host disease (GVHD) after hematopoietic stem cell transplantation (Trzonkowski et al. 2009a). The treatment resulted in improvement patients’ condition and allowed lowering glucocorticoid doses. What is more, no significant side effects were observed. At present, most clinical trials based on infusion of ex vivo expanded tTreg cells have been performed in the prophylaxis or treatment of GVHD but the Treg cell transfer may be also beneficial in the treatment of some autoimmune diseases: type I diabetes, myasthenia gravis and lupus erythematosus (Gliwiński et al. 2017). However, to obtain clinically meaningful effects, high number of tTreg cells for infusion is necessary. Since tTreg cells constitute a minor fraction of peripheral blood CD4^+^ T cells and they are characterized by low proliferative index, there are some problems with obtaining the final product of high purity (Gliwiński et al. 2017). Moreover, only some of sorted cells are efficient suppressors (Trzonkowski et al. 2009b). Furthermore, some studies have demonstrated tTreg instability and dysfunction in the inflammatory condition following infusion (Lan et al. 2012; Lu et al. 2014).

To overcome the mentioned above obstacles, attempts are being made to generate in vitro iTreg cells in sufficient number from conventional CD4^+^ T cells, using for example rapamycin or all-trans retinoic acid (Candia et al. 2017). This cells could mimic peripheral Treg cells and may be especially beneficial in hyperactive immune pathologies including chronic inflammatory diseases associated with tissue destructions.

In the present study we verified the ability of various pharmacological agents with suggested modulatory effect on Treg cell development/activity: rapamycin (Fanigliulo et al. 2015; Li et al. 2014), prednisolone (Karagiannidis et al. 2004; Mathian et al. 2015), atorvastatin (Forero-Peña and Gutierrez 2013), inosine pranobex, sodium butyrate (Fontenelle and Gilbert 2012), and glatiramer acetate (Haas et al. 2009; Hong et al. 2005) to facilitate/augment generation of iTreg cells in standard in vitro cultures, with the aim to further investigations for clinical application.

## Materials and Methods

### Human T Cell Isolation

Peripheral blood mononuclear cells (PBMCs) were prepared from buffy coats obtained from the Regional Centre of Blood Donation and Blood Therapy (Warsaw, Poland). The cells were isolated by Ficoll-Hypaque (Histopaque-1077, Sigma-Aldrich, St. Louis, MO, USA) density gradient centrifugation. After washing in phosphate-buffered saline (PBS), PBMCs were counted and suspended in culture medium [RMPI medium supplemented with antibiotics (penicillin, 100 U/ml and streptomycin, 0.1 g/L) + antimycotic (Amphotericin B, 0.25 mg/L) (Sigma-Aldrich) and 10% heat-inactivated fetal bovine serum (Gibco-Invitrogen, Grand Island, NY, USA)], aliquoted in cryotubes, frozen and transferred to liquid nitrogen. CD4^+^ T cells were isolated from PBMCs before experiments by a negative selection method, using magnetic-activated cell sorting (MACS) system (CD4^+^ T cell isolation kit; MiltenyiBiotec, BergischGladbach, Germany). The purity of isolated CD4^+^ cells was assessed using flow cytometry and confirmed to be > 96%.

### iTreg Differentiation

Following MACS separation, CD4^+^ T cells were cultured in 24 flat-bottom well plates at a concentration of 2 × 10^5^ cells/well in culture medium. For Tregs induction, cells were stimulated with CD3/CD28 beads (0.25 × 10^5^/ml) (Dynabeads Human T-activator, Thermo Fisher Scientific, Waltham, MA, USA), in the presence of TGF-β1 (2 ng/ml) (PeproTech, London, UK). To prevent proliferation of tTreg cells with high expression of CD25, interleukin (IL)-2 was not added to cultures. The following agents were tested for their action on Treg cell development: sodium butyrate (Sigma Aldrich) (final concentrations: 4, 20, 100, 500 µM), glatiramer acetate (final concentrations: 1, 5, 25, 125 µg/ml), inosine pranobex (Gedeon Richter Polska, Poland) (final concentrations: 50, 100, 200 mg/ml), rapamycin (Sigma Aldrich) (final concentrations: 4, 20, 100, 500, 2500 ng/ml), prednisolone (Sigma Aldrich) (final concentrations: 2.5, 25, 250, 2500, 25,000 ng/ml), atorvastatin (final concentrations: 1.6, 8, 40, 200, 1000 ng/ml) or acetic acid (final concentrations: 4, 20, 100, 500 µM). The cells were incubated for 5 days, followed by FACS analysis.

### Flow Cytometry and Antibodies

The phenotype of CD4^+^ T cells was determined based on CD25 and Foxp3 markers. Surface antigens CD4 and CD25 were stained with anti-CD4-PerCP (clone SK 3, BD Biosciences, USA) and anti-CD25-FITC (clone M-251; BD Biosciences) monoclonal antibodies in the dark for 20 min at 4 °C. Following staining, the cells were washed twice with PBS and stained intracellularly with Foxp3-PE Staining Kit (clone PCH101; eBioscience, USA) according to manufacturer’s protocol. Acquisition was performed on BD Accuri C6 (BD Biosciences). FACS data were analyzed using FlowJo.

### Functional Testing of Treg Inhibitory Activity in MLR

To evaluate the in vitro immunosuppressive activity of induced Treg cells (iTreg), mixed lymphocyte reaction (MLR) was performed. Following 5 days of iTreg differentiation, the cells were isolated from cultures using MACS system (T regulatory cell isolation kit; MiltenyiBiotec, BergischGladbach, Germany). The purity of isolated Treg cells was assessed using flow cytometry and confirmed to be > 96%. Isolated iTregs rested for 2 days in culture medium with addition of IL-2 (50 IU/ml) (Proleukin; Novartis, Switzerland). In the next step, freshly isolated allogenic PBMCs were inactivated by gamma irradiation for 90 min and served as stimulators (PBMC-stim). In the assay, 1 × 10^5^ stimulatory cells were incubated with 1 × 10^5^ responder CD4^+^ T cells (Tresp) and co-cultured with 0.5 × 10^5^, 0.25 × 10^5^, or 0.125 × 10^5^ autologous regulatory T cells. Cells were cultured in 96-well flat-bottom plates at 37 °C in a humidified atmosphere with 5% CO_2_. After 5 days, cultures were pulsed with 1 μCi/well of ^3^H-thymidine (113 Ci/nmol, NEN, Life Science Products, Zaventem, Belgium) for the last 18 h of the incubation and harvested with an automated cell harvester (Skatron). The amount of ^3^H-thymidine incorporated into the proliferating cells was measured using a Wallac Microbeta scintillation counter (Wallac), giving the level of radioactivity as “corrected counts per minute” (cpm). Experiments were performed in triplicates.

### Cytokines

Cytokine concentrations in supernatants were measured using a LEGENDplex™ bead-based cytokine kit (BioLegend, USA), according to the manufacturer’s protocol. On day 0, cells were plated in 24 flat-bottom well plates at a concentration of 8 × 10^5^ cells/0.8 ml/well supplemented with CD3/CD28 beads, TGF-β1 (see “iTreg Differentiation” section), and prednisolone or rapamycine. On day 5, supernatants were discarded and replaced with fresh culture medium. After 2-day resting period culture supernatants were harvested, centrifuged, and kept frozen until the cytokine analysis procedure.

### Analysis of Stability of Induced Treg Cells

After 5 days of Treg induction in the presence of either prednisolone or rapamycin (see “iTreg Differentiation” section), culture medium was discarded and replaced with medium containing 50 U/ml IL-2 (Proleukin, Novartis). The cells were then incubated for the next 4 days followed by FACS analysis of Treg markers.

### Statistical Analysis

Differences between samples with or without addition of the same immunomodulator were examined using paired Student’s *t* test. Differences between samples with or without addition of different immunomodulator in mixed lymphocytes reaction assay was examined using unpaired Student’s *t* test. *P* smaller than 0.05 was considered as significant.

## Results

### Induction of CD4^+^CD25^high^Foxp3^high^ Treg Cells

**Fig. 1:**
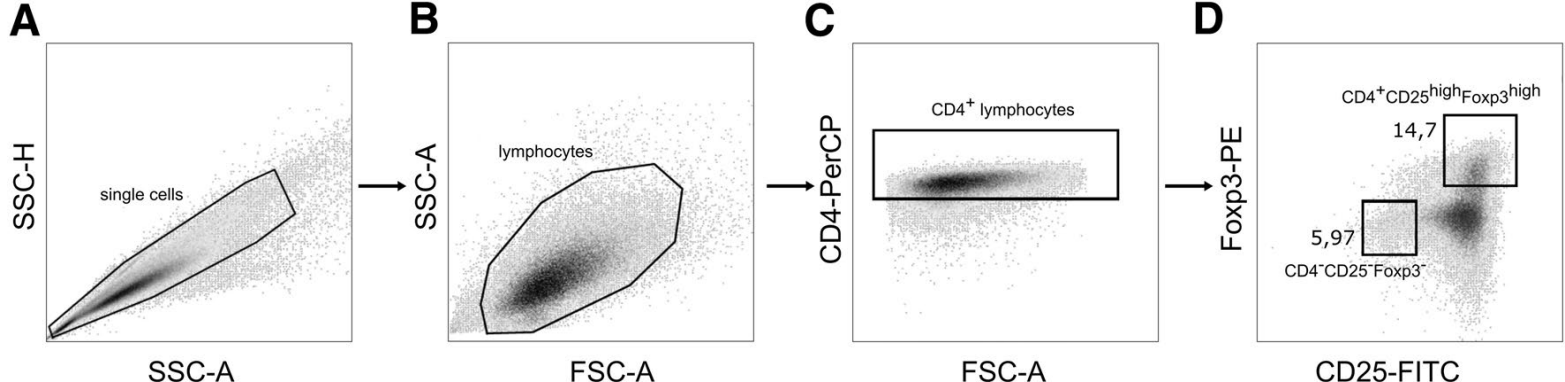
Identification of induced Treg cells. **a** Treg cells were generated from CD4^+^ T cells incubated with CD3/CD28 beads and TGF-β for 5 days. The cells were analyzed by FACS. **b** Primary gate was set on lymphocytes depending on their forward and side scatter properties. **c** Secondary gate was set on CD4^+^ T lymphocytes. **d** Treg cells were identified using anti-CD25 and anti-Foxp3 antibodies as CD4^+^CD25^high^Foxp3^high^ cells. Numbers show percentage of gated cells

In preliminary experiments, optimal conditions for Treg cell differentiation were established: CD4^+^ T cells were stimulated by CD3/CD28 beads in the presence of TGF-β. The number of Treg cells (recognized as CD4^+^CD25^high^Foxp3^high^ cells), as well as expression of Foxp3, was the highest in the fifth day of incubation. The dose of CD3/CD28 beads was adjusted so that differentiating CD4^+^ T lymphocytes were not overstimulated (CD3/CD28 beads to T cell ratio 1:8). Similarly, the dose of TGF-β (2 ng/ml) was suboptimal. We assumed that high doses of TGF-β could lead to maximum level of differentiation of CD4^+^ T cells to Tregs, which could prevent further augmentation by examined immunomodulators. Identification of iTreg cells is presented in Fig. 1.

### Stimulation of CD4^+^ T cells in Presence of Rapamycin, Prednisolone, Glatiramer Acetate, Sodium Butyrate or Inosine Pranobex Leads to Increased Proportion of Treg Cells in Cultures

**Fig. 2:**
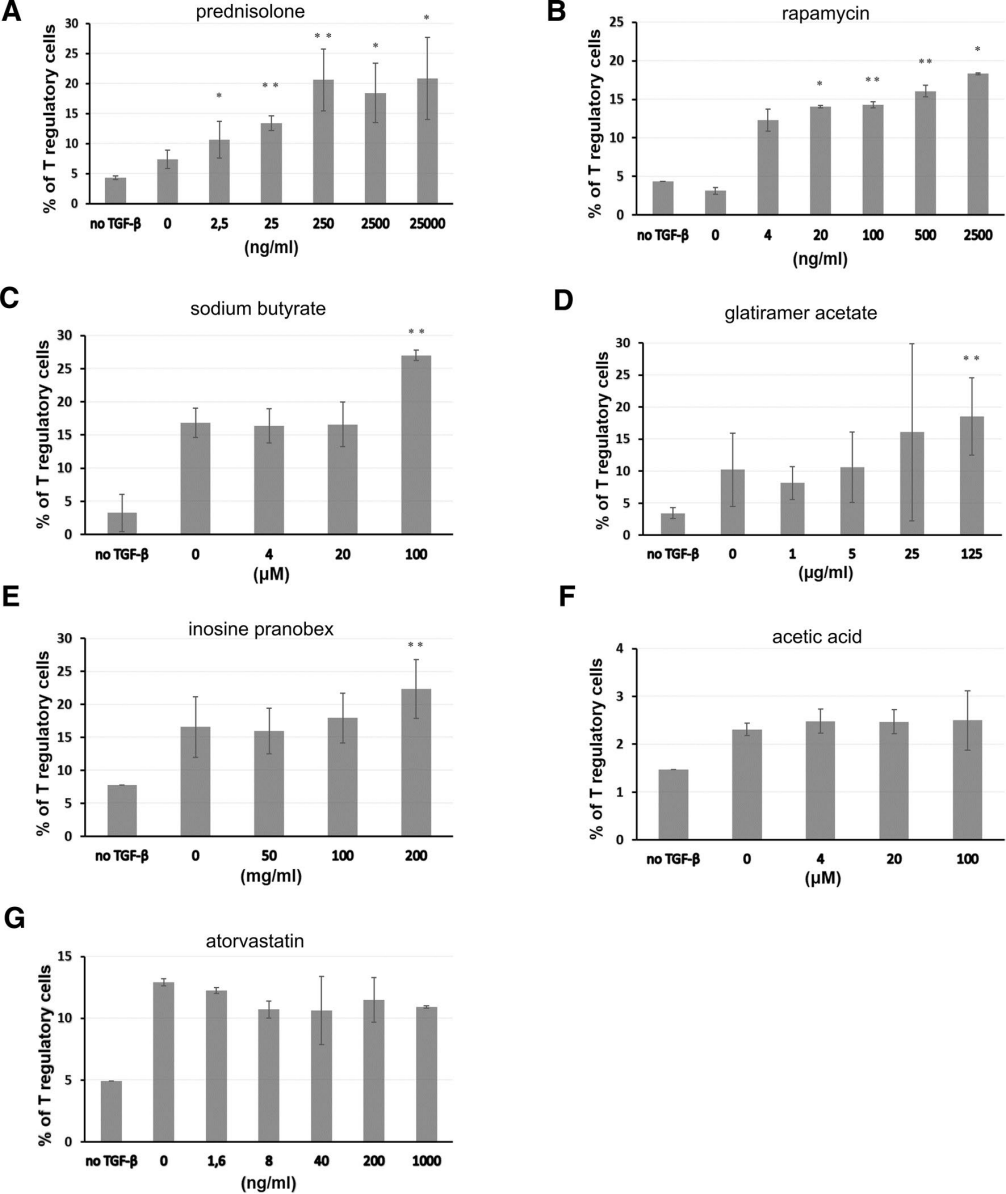
Frequency of Treg cells in cultures with different immunomodulators. Cells were analyzed following 5 days of culture with CD3/CD28 beads, TGF-β and immunomodulators (for details, see “Materials and Methods”). CD4^+^ T lymphocytes were co-cultured with prednisolone (**a**), rapamycin (**b**), sodium butyrate (**c**) glatiramer acetate (**d**), inosine pranobex (**e**), acetic acid (used as a negative control for butyrate) (**f**), and atorvastatin (**g**). *p < 0.05, **p < 0.01 vs. culture with no immunomodulator

**Fig. 3:**
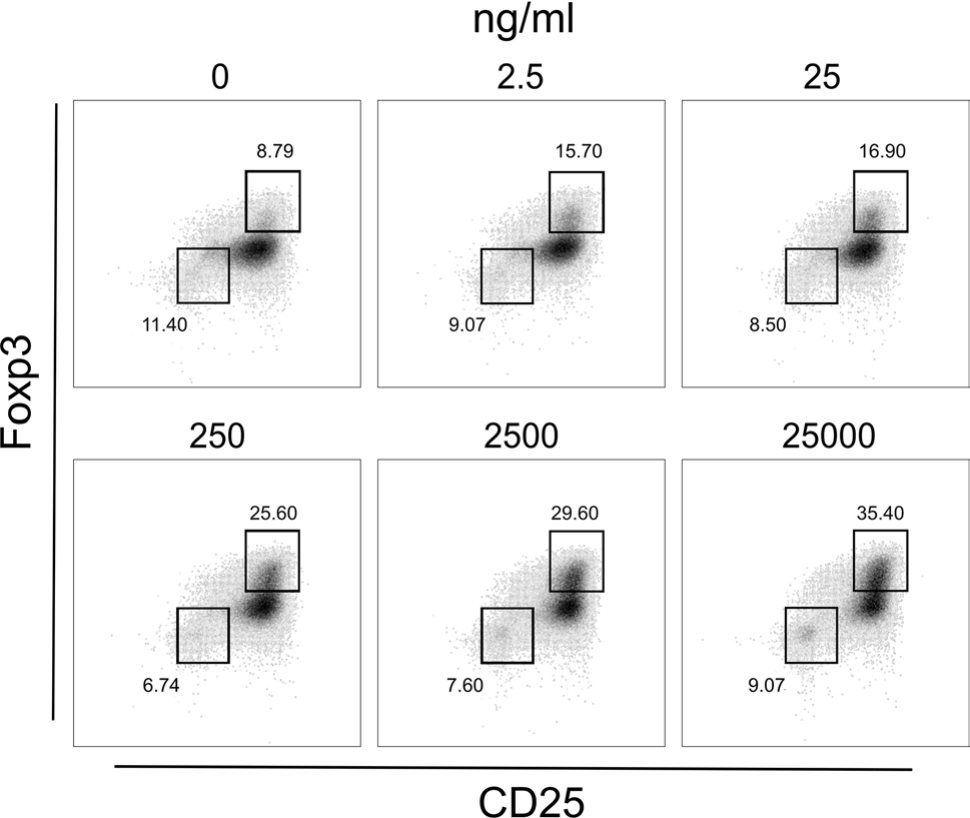
Frequency of Treg cells in cultures with increasing concentrations of prednisolone. CD4^+^ T cells were incubated with CD3/CD28 beads, TGF-β and increasing doses of prednisolone. Cells were analysed after 5 days of incubation (for details, see “Materials and Methods”). Gates were set on CD4^+^CD25^–^Foxp3^–^ and CD4^+^CD25^high^Foxp3^high^ cells. Results are representative for three independent experiments

First, we investigated the ability of a panel of immunomodulatory agents to increase differentiation of CD4^+^ T cells incubated with CD3/CD28 beads and TGF-β to CD4^+^CD25^high^Foxp3^high^ cells. Co-culture of CD4^+^ T lymphocytes with immunomodulators resulted in an increase of Tregs in comparison to TGF-β alone, in a dose-dependent manner. The strongest effect was observed in cultures with prednisolone concentration of 250 ng/ml and more (Fig. 2a, representative FACS graphs are presented in Fig. 3), rapamycin (4 ng/ml and more, Fig. 2b), sodium butyrate (20 and 100 µM, Fig. 2c), glatiramer acetate (125 ng/ml, Fig. 2d), and inosine pranobex (200 mg/ml, Fig. 2e). In comparison with rapamycin, prednisolone was found to inhibit proliferation of CD4^+^ T cells (stimulated with CD3/CD28 beads in the presence of TGF-β) to a much lesser extent. Significantly lower number of cells was observed in cultures with the highest concentration of prednisolone (25 μg/ml), while rapamycin decreased number of cells already at a dose of 100 ng/ml (and higher) (Table 1). Incubation of lymphocytes with atorvastatin or acetic acid (used as a control for butyrate) did not lead to significant increase in proportion of Treg cells (Fig. 2f, g).

**Table 1:** Recovery of lymphocytes in stimulated cultures incubated with prednisolone or rapamycin

CD3/CD28 beads	TGF-β	Immunomodulator	Number of cells × 10^5^ (mean ± SD)
+	+	Prednisolone	
		25 μg/ml 2.5 μg/ml 0.25 μg/ml	1.52 ± 0.13* 1.79 ± 0.44 1.78 ± 0.43
+	+	Rapamycin	
		2500 ng/ml 500 ng/ml 100 ng/ml	1.28 ± 0.17** 1.31 ± 0.30** 1.48 ± 0.32**
+	+	–	2.42 ± 0.38
+	−	–	2.99 ± 0.54

CD4^+^ T cells (2 × 10^5^) were stimulated for five days with CD3/CD28 beads (supplemented with TGF-β) in the presence of prednisolone or rapamycin

*p < 0.05, **p < 0.01, vs. cell culture stimulated with CD3/CD28 beads supplemented with TGF-β

Induction of Treg cells in the presence of the tested agents was also associated with augmented expression of Foxp3 (apart from atorvastatin and acetic acid). The expression of Foxp3 was the highest in CD4^+^ T cells incubated with prednisolone and rapamycin. High concentrations of prednisolone (25 μg/ml) and rapamycin (500 ng/ml) resulted in a 2.11-fold and 2.38-fold, respectively, increase in Foxp3 median florescence intensity (MFI) compared with control samples (CD4^+^ T lymphocytes stimulated with CD3/CD28 beads in the presence of TGF-β) (Table 2).

**Table 2:** Median Foxp3 expression (MFI) in Treg cells induced in cultures with different immunomodulatory agents

Immunomodulator	Foxp3 MFI in cultures with immunomodulator (median ± SD)	Foxp3 MFI in controls (median ± SD)	Foxp3 MFI index^a^
Prednisolone			
25 μg/ml	7643 ± 411		2.11**
2.5 μg/ml	6131 ± 724		1.69**
0.25 μg/ml	5847 ± 557	3622 ± 140	1.61***
Rapamycin			
500 ng/ml	8374 ± 780		2.38*
100 ng/ml	7689 ± 188	3525 ± 57	2.18*
Sodium butyrate (100 μM)	11,962 ± 6540	7741 ± 3372	1.55*
Glatiramer acetate (125 μg/ml)	13,437 ± 8574	6901 ± 3512	1.95
Inosine pranobex (200 mg/ml)	7699 ± 1540	5925 ± 609	1.30*
Acetic acid (100 μM)	3647 ± 313	3718 ± 146	0.98
Atorvastatin (1 μg/ml)	5080 ± 62	5326 ± 184	0.95

Foxp3 expression was assessed in cultures of CD4^+^ T cells following 5-day stimulation (CD3/CD28 beads + TGF-β) in the presence of immunomodulatory agents

*p < 0.05, **p < 0.01, ***p < 0.001

^a^Foxp3 index represents Foxp3 immunomodulator to Foxp3 control ratio

### Treg Cells Generated in the Presence of Prednisolone and Rapamycin are Suppressive in a Functional Assay

**Fig. 4:**
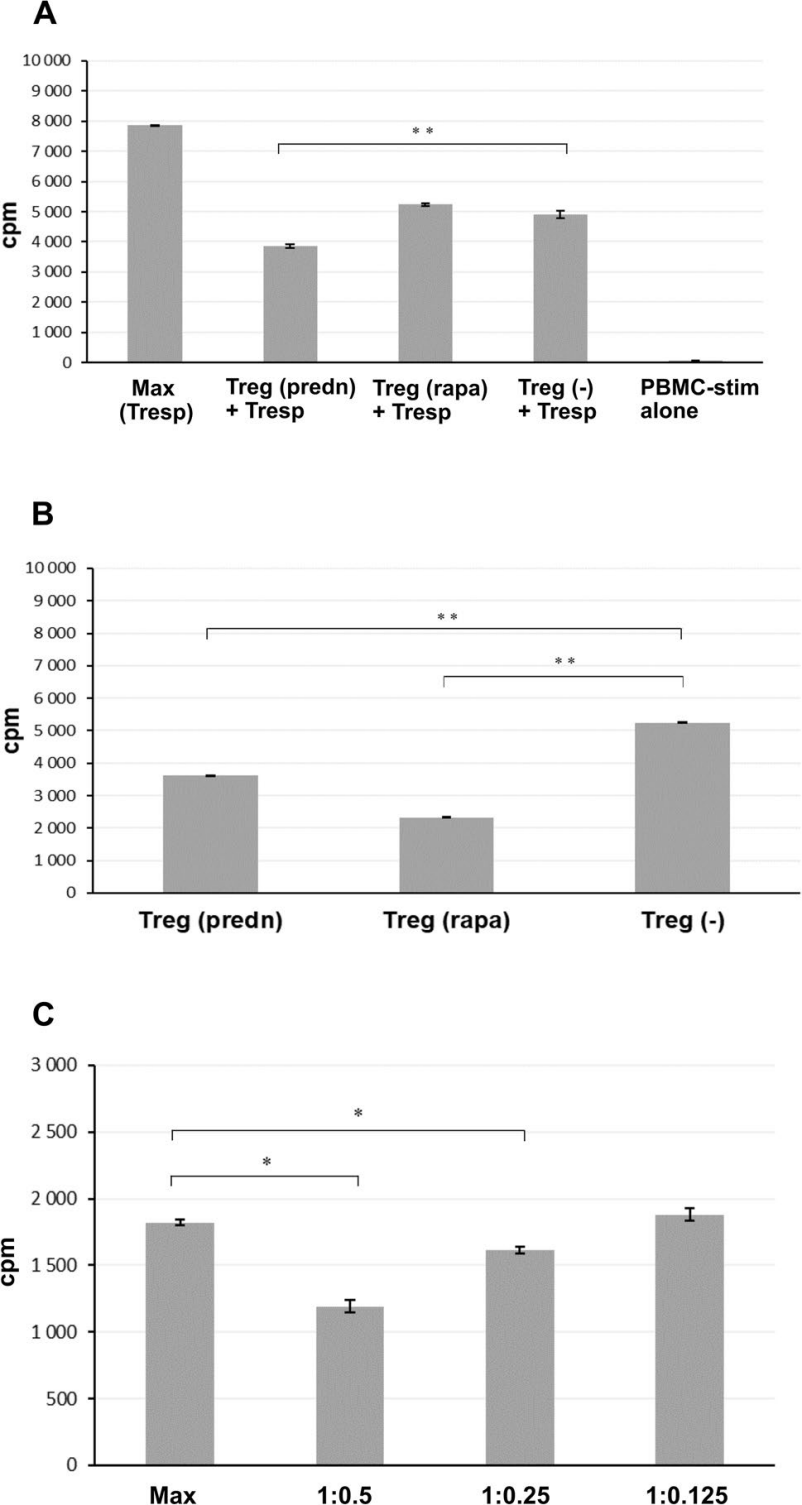
**a** Suppression of allogeneic mixed lymphocyte reaction (MLR) by Treg cells generated in the presence of prednisolone or rapamycin. Tregs induced in 5-day cultures from CD4^+^ T cells incubated with CD3/CD28 beads and TGF-β in the presence of prednisolone (250 ng/ml) or rapamycin (100 ng/ml), as described in Materials and Methods, were incubated with allogeneic peripheral blood mononuclear cells (PBMC) (gamma-irradiated, PBMC-stim) and autologous responding CD4^+^ T cells (Tresp) for 5 days (to give PBMC-stim: Tresp: Treg ratio 1:1:0.5. 18 h before the end of incubation, 1 μCi ^3^H-thymidine was added to each well. At the end of the culture, cells were harvested and maximal count per minute (cpm) was measured. For clarity, proliferation of Treg cells was subtracted. Results are representative for two independent experiments. Max (Tresp)—cpm in cultures of autologous responding CD4^+^ T cells incubated with gamma-irradiated allogeneic PBMC; Treg (predn), Treg (rapa), Treg (−)—Treg cells induced in the presence of prednisolone, rapamycin, and with no agent, respectively; Treg (predn) + Tresp—cultures of autologous responding CD4^+^ T cells incubated with stimulatory allogeneic PBMC supplemented with Treg cells that were induced in the presence of prednisolone; PBMC-stim alone—cpm in cultures of gamma-irradiated stimulatory allogeneic PBMC alone. **b** Proliferation of Treg cells incubated with allogeneic PBMC-stim cells (gamma-irradiated) (PBMC-stim: Treg ratio 1:0.5). **c** Proliferative response of autologous responding T CD4^+^ cells in allogeneic MLR in the presence of different number of Treg cells (Tresp: Treg ratio 1:0.5, 1:0.25, and 1:0.125) generated in cultures with prednisolone. *p < 0.01, **p < 0.001

Activation of CD4^+^ T cells in the presence of TGF-β significantly increased number of Treg cells and most of the tested drugs/compounds enhanced this effect. Prednisolone and rapamycin seemed the most effective in this activity (Figs. 2, 3). However, it was interesting if the induced Treg cells were functional. To test this hypothesis, activity of Treg cells generated in the presence of prednisolone or rapamycin were investigated in allogeneic MLR. As shown in Fig. 4, Treg cells from cultures incubated with prednisolone or rapamycin effectively suppressed proliferation of allo-stimulated T cells in MLR. Interestingly, while Treg cells generated in cultures with rapamycin showed similar suppressive activity when compared to control cultures, prednisolone-activated Treg cells were found superior and exerted significantly stronger suppressive effect (Fig. 4a). This effect could be related, in part, to higher number of Treg cells in cultures with prednisolone since higher rate of proliferation was observed in Treg cultures stimulated by allogeneic cells in the presence of prednisolone than with rapamycin (Fig. 4b). Certainly, an analysis of immunosuppressive cytokines and iTreg stability (e.g., assessment of Treg-specific demethylation region—TSDR), in prednisolone cultures could support superiority of prednisolone over rapamycin. Prednisolone-generated Treg cells exerted measurable inhibitory effect in MLR at 1:0.5 but also at 1:0.25 Tresp-to-Treg cell ratio (Fig. 4c).

### Prednisolone and Rapamycin Prolong Phenotypic Stability of iTreg Cells

To effectively control inflammation and excessive immune response, Treg stability should be maintained over time even in the absence of Treg-promoting stimuli. To answer the question if the Treg-promoting effect of prednisolone and rapamycin is transient or permanent, we cultured the cells after 5 days of Treg induction for additional 4 days in the presence of IL-2 only. As presented in Table 3, in contrast to a significant loss of regulatory phenotype of cells activated only in the presence of TGF-β, percentage of CD4^+^ T cells expressing Treg markers (CD25^high^Foxp3^high^) was much higher in resting cultures primarily activated with addition of either prednisolone or rapamycin. Treg-promoting effect of prednisolone is especially notable, since a proportion of Treg cells was preserved in a 4-day resting cultures of cells primarily generated in the presence of prednisolone even when no IL-2 was added to cultures (5.5% vs. 1.21%, when compared to primary activation with TGF-β only). However, during a 4-day resting period, expression of Foxp3 decreased significantly, both in Treg cells that were primarily induced with TGF-β only and in cells co-incubated with either prednisolone or rapamycin (data not shown).

**Table 3:** Effect of prednisolone and rapamycin on stability of in vitro-induced Treg cells (CD25^high^Foxp3^high^CD4^+^ T lymphocytes)

	Immunomodulator supplemented	CD25^high^Foxp3^high^ CD4^+^ T lymphocytes (%) (mean ± SD)	
Day + 5 (incubation of CD4 T cells with CD3/CD28 beads + TGF-β in the presence or absence of immunomodulators)	None	7.5 ± 0.01	
	Prednisolone:		
	2.5 μg/ml 0.25 μg/ml	15.4 ± 0.02 8.4 ± 0.00	
	Rapamycin:		
	500 ng/ml 100 ng/ml	12.1 ± 0.01 8.2 ± 0.01	
		+ IL-2	− IL-2
Day + 9 (prolonged incubation on days 5–9 with or without IL-2, with no CD3/CD28 beads, no TGF-β, and no immunomodulators)	Culture conditions on days 0–5:		
	None	2.1 ± 0.00	1.21 ± 0.01
	Prednisolone:		
	2.5 μg/ml 0.25 μg/ml	7.5 ± 0.00 6.5 ± 0.01	5.5 ± 0.01 2.6 ± 0.00
	Rapamycin:		
	500 ng/ml 100 ng/ml	6.1 ± 0.00 3.6 ± 0.01	2.8 ± 0.00 1.5 ± 0.00

CD4^+^ T lymphocytes were analyzed following five days of culture with CD3/CD28 beads, TGF-β, and in the presence or absence of immunomodulators (for details, see “Materials and Methods”) (day + 5, upper part of the table). On the day 5, culture medium was replaced with medium containing either IL-2 or no cytokine and the cells were analyzed following prolonged four-day incubation (day + 9, lower part of the table). Samples were tested in duplicates

### Effects of Prednisolone and Rapamycin on Cytokine Production in Cultures of iTreg Cells

Function of Treg cells is mediated either by the direct cell-to-cell contact or via cytokines, preferentially IL-10 and TGF-β. The cells, however, may produce and release other cytokines. In fact, Treg cells are plastic and may be converted into Th17 subpopulation in the presence of proinflammatory cytokines. To investigate if generation of iTreg cells in the presence of prednisolone or rapamycin may shape/change their cytokine-production profile, we measured levels of IL-10 (representative immunosuppressive cytokine), IL-6 and tumor necrosis factor (TNF)-α (characteristic proinflammatory cytokines) in respective cultures. As shown in Table 4, neither prednisolone nor rapamycin significantly affected the ability of induced Treg cells to produce IL-10 and TNF-α. What is interesting, despite the large interindividual variation in cytokine production, iTreg generated in the presence of prednisolone seemed to produce smaller amounts of IL-6 in comparison with activated cultures incubated with no TGF-β or TGF-β alone. This observation can have clinical translation, since IL-6 is recognized as the key cytokine promoting conversion of Tregs into Th17 cells (Schmitt and Williams 2013) and, as shown in recent studies by Fu et al. (2017), prednisone inhibits the ability of proinflammatory cytokines to stimulate differentiation of decidual immune cells into Th17 cells.

**Table 4:** Effects of prednisolone and rapamycin on cytokine production in cultures of iTreg cells

Incubation conditions	Concentration of cytokines in supernatants (pg/ml) (mean ± SD) (*n* = 3)		
	IL-10	IL-6	TNF-α
CD3/CD28 beads	20.1 ± 10.5	51.5 ± 22.7	66.3 ± 20.5
+ TGF-β	17.5 ± 7.7	67.1 ± 43.4	100.1 ± 31.1
+ TGF-β + prednisolone (2.5 μg/ml)	15.7 ± 7.5	3.4 ± 1.4	22.3 ± 13.4
+ TGF-β + prednisolone (0.25 μg/ml)	27.1 ± 8.5	11.1 ± 6.2	43.5 ± 16.6
+ TGF-β + rapamycin (500 ng/ml)	11.7 ± 3.6	73.7 ± 46.1	174.9 ± 71
+ TGF-β + rapamycin (100 ng/ml)	21.2 ± 17.2	98.8 ± 93.4	307.4 ± 199.7

iTreg cells were induced in the presence of prednisolone or rapamycin for 5 days, as described in “Material and Methods”. On day 5, culture supernatants were discarded and replaced with fresh culture medium. After 2-day resting period supernatants were harvested, centrifuged, and kept frozen until the cytokine analysis procedure

## Discussion

In the present study we compared the ability of different pharmacological compounds (drugs), with suggested and real immunomodulatory activity, to promote generation of Treg cells from conventional CD4^+^ T cells. In the literature, there is a lack of complex comprehensive analysis of the immunoregulatory potential of the compounds. We showed that rapamycin, prednisolone, sodium butyrate, glatiramer acetate and inosine pranobex, more or less efficiently and usually in a dose-dependent manner, increased the number of iTreg cells in cultures of activated CD4^+^ T cells in the presence of TGF-β. The highest percentage of CD4^+^CD25^high^Foxp3^high^ lymphocytes appeared in cultures with prednisolone and rapamycin. However, it remains the open question if this high percentage resulted more from a direct promotion of Treg cells than from inhibition of non-Treg cell proliferation. In our study, atorvastatin and acetic acid neither exerted significant effects on induction of T regulatory cells nor affected expression of Foxp3.

Results of our studies shed light on mechanisms of the immunomodulatory effect of some tested compounds, explained discrepancies observed in studies of different authors and even demonstrated unpredictable effects. To induce Treg cells, in our experimental protocol TGF-β was used in suboptimal concentration (2 ng/ml) and combined with immunomodulators that we could expect to enhance the TGF-β effect. Results of the study could answer the cardinal clinical question: whether immunomodulators/drugs can augment physiological effect of TGF-β in organs (compartments) in which this cytokine is produced in not high amounts.

Statins (including atorvastatin) are pharmacological inhibitors of the activity of 3-hydroxy-3-methyl-glutaryl-CoA reductase. Apart from inhibition of cholesterol synthesis, the drugs exert mild anti-inflammatory effect. This effect, in part, is dependent on Treg cells (Forero-Peña and Gutierrez 2013). Atorvastatin (but not mevastatin nor pravastatin) was shown to increase number of Treg cells in vitro (Mausner-Fainberg et al. 2008). On the contrary, Guasti et al. (2016) showed that atorvastatin (even in very high concentrations—5 μM) did not affect PHA-induced Treg cells (Foxp3^+^). Our studies are in agreement with the other study. The discrepancy between our studies and those by Mausner-Fainberg et al. (2008) can be explained in that they used much higher concentrations of atorvastatin, in non-stimulated cultures and, in fact, they tested the effect of the drug on natural tTreg cells.

Metabolites produced by gut microbiota (including short-chain fatty acids, such as butyric acid) in mice promote generation of pTreg cells and, jointly with direct anergizing effects on effector T cells, contribute to immunological homeostasis in the gut (Arpaia et al. 2013; Fontenelle and Gilbert 2012; Whibley et al. 2019). We confirmed the effect of butyrate on development of Treg cells in human CD4^+^ T cells and, in contrast to the study of Schmidt et al. (2016), we showed that the presence of IL-2 in culture is not obligatory in this phenomenon.

Glatiramer acetate (GA) is a pharmacological immunomodulatory agent approved for treatment of relapsing–remitting multiple sclerosis (Mitsikostas and Goodin 2017). Mechanisms of action of this drug have not been fully elucidated. Recent studies have demonstrated restoration of Treg cell function (CD4^+^CD127^low^CD25^high^) in patients following long-term treatment with GA (Spadaro et al. 2017). This effect seems to result from expansion of thymic Treg cells (Haas et al. 2009). Our studies, showing both increased number of cells with Treg characteristic and increased expression of Foxp3 marker in T cells incubated in the presence of GA (Fig. 2d, Table 2), demonstrate that inducible Treg cells may contribute to the overall therapeutic effect of this immunomodulator in patients with multiple sclerosis.

The original finding of our studies is showing the promoting effect of inosine pranobex (IP) (isoprinosine, inosine dimepranol acedoben) on Treg cell development. The agent is recognized as a mild immunostimulator, both in vitro and in vivo, with a direct antiviral activity (Campoli-Richards et al. 1986; Majewska et al. 2015). In our recent studies, in cultures of human lymphocytes, IP stimulated production of cytokines characteristic for Th1 response: interferon-γ and TNF-α while inhibiting typical immunosuppressive cytokine IL-10 (Lasek et al. 2015). We cannot offer simple explanation of this paradoxical effects of IP on immune mechanisms. Maybe, the cytokine milieu during T cell activation is the clue. Systemic in vivo stimulation of effector T lymphocytes and general immunostimulatory effect of IP does not exclude its local effect on promotion of pTreg (in the presence of high concentrations of TGF-β in the local environment).

Treg-enhancing effects of rapamycin and prednisolone deserve separate comments. Rapamycin is mammalian target of rapamycin inhibitor displaying strong immunosuppressive activity, arresting the cell cycle in G1 phase without blocking production of IL-2 (Li et al. 2014). The drug was found effective in expansion of CD4^+^CD25^+^Foxp3^+^ natural regulatory (tTreg) cells more than decade ago, and is being used in protocols aimed at expansion of polyclonal Treg cells for therapeutic purposes (Alsuliman et al. 2016; Mathew et al. 2018). In our studies we evidenced that rapamycin may effectively promote development of polyclonal iTreg cells that, as suggest our results of a functional assay of their activity in MLR (Fig. 4), could be used in organ transplantation. In contrast to rapamycin, glucocorticoid (GC) effects on cellular regulatory mechanisms have not been investigated sufficiently. GCs are potent anti-inflammatory and immunosuppressive agents that are widely used in autoimmune, inflammatory, and allergic diseases (Coutinho and Chapman 2011). Intuitively, their application should positively affect Treg cell number and/or function in these disorders but results of in vivo studies have been inconclusive: both increased (Mathian et al. 2015; Seissler et al. 2012) and decreased (Olsen et al. 2015; Stock et al. 2005) Treg cell activity, as well as no effect on these cells (Sbiera et al. 2011) were documented following GC treatment. This inconsistencies may result from doses of GC used, time of patients’ treatment, type of pathology and initial level of Treg cells, source of cells (peripheral blood vs. locally infiltrating cells), definition of Treg subset etc. Surprisingly, no complex in vitro studies concerning this interesting subject have been performed. The only reliable in vitro study of GC in the context of regulatory T cells in humans demonstrated induction of Foxp3 and IL-10 but not TGF-β mRNA expression in human CD4^+^ T cells in 6 and 24 h cultures stimulated with anti-CD2/3/28 antibodies in the presence of IL-2 (Karagiannidis et al. 2004). The cells may be recognized as Tr1 regulatory cells. In our studies we have unequivocally shown the effect of prednisolone on generation of inducible in vitro Treg cells and, what is interesting, the cells were found to have at least as potent immunosuppressive potential as rapamycin in a functional assay (Fig. 4). The open question is if the similar effect of GC would occur in pure cultures of tTreg cells but some data points on selective effect of GC on pTreg cell development from conventional T cells. As shown by Bereshchenko et al. (2014), GCs stimulate expression of glucocorticoid-induced leucine zipper protein. This protein enhances TGF-β signaling by binding to and promoting phosphorylation of Smad2 and activation of Foxp3 expression. Certainly, in the next step of our study aimed at investigation of the effect of prednisolone on generation of Treg cells from naïve CD4^+^CD25^–^ T cells we will resolve this problem.

It is worth noting that steroids exert proapoptotic effect on T lymphocytes in many settings (Coutinho and Chapman 2011). Indeed, we observed a meaningful inhibitory effect of prednisolone on the number of lymphocytes in stimulated T cell cultures incubated with TGF-β but only in cultures incubated with the highest concentrations of prednisolone. In fact, recovery of lymphocytes for FACS analysis was higher in prednisolone than rapamycin-cultivated CD4^+^ T cells (Table 1).

Another question is if iTreg-inducing effect of prednisolone which is observed in vitro can be translated in vivo. Since concentration of prednisolone in serum up to 1 μg/ml was detected following single oral administration of the drug (Gai et al. 2005), it is highly probable that immunomodulatory effect of glucocorticoids on Treg cells may appear in vivo.

Sustained Foxp3 expression is necessary for immunosuppressive cell activity (Gao et al. 2012). However, this transcription factor may be transiently expressed in a high percentage of activated T cells after in vitro stimulation of human CD4^+^CD25^–^cells (Wang et al. 2007). Such expression is associated with low responsiveness of these cells, but they lack significant suppressive capacity. Nevertheless, Tregs express higher level of Foxp3 than activated CD4^+^CD25^–^ cells. Thus determination of MFI of Foxp3 should be used as a way to discriminate the cells having suppressive abilities from activated CD4^+^CD25^+^ T cells devoid of regulatory potential.

Other issue is the functional stability of in vitro-induced Tregs. At present, the most reliable way to show Treg stability is verification of demethylation pattern in TSDR. Although TSDR in tTregs is demethylated, in vitro induction of iTregs with TGF-β does not lead to demethylation of TSDR (Ohkura et al. 2012). Nevertheless, functionality of in vitro induced iTreg cells was shown both in vitro and in vivo (Hippen et al. 2011). Addition of some agents to Treg-inducing protocols may increase their function in vivo even to greater extent (Lee et al. 2012). Results of our studies are in agreement with the latter one, showing clearly that rapamycin and especially prednisolone, when present during Treg-inducing phase, can promote development of higher number of Treg cells. However, the matter is unknown how long these iTreg cells will retain their suppressive activity in vivo.

In summary, our studies have shown different potential of a panel of immunomodulatory pharmaceutical agents to promote development of Treg cells. Our findings may be helpful in developing the most effective protocols of T regulatory lymphocytes’ induction. The most interesting finding of our studies is showing unequivocal and strong Treg-inducing effect of prednisolone. This effect may contribute to immunosuppressive and anti-inflammatory property of glucocorticoids observed in vivo. Since prednisolone seems as optimal as rapamycin in stimulating iTreg cells, preparation of prednisolone-generated Treg cells could be considered as an option for adoptive cellular treatment.
